# Physical Methods in Deformity and Disease: Some Hindrances to Their More Extended Employment

**Published:** 1917-09-01

**Authors:** 


### PHYSICAL METHODS IN DEFORMITY & DISEASE.

### Some Hindrances to their More Extended Employment.

IN our issues of August 11 and 18 we had the pleasure of publishing an address on "Physical Orthopædics" by Dr. R. Fortescue Fox, and in another column of the present number we print a letter, dealing with the same subject, from Mr. French, of Northampton. The address includes the opinions of an expert physician who has given prolonged and careful attention to the subject on which he speaks. Mr. French writes from a different standpoint. He pretends to no knowledge of scientific medicine, but he has for a number of years had practical experience in the application of various physical methods of treatment to persons suffering from deformities or disease. Both contributions agree in raising the question, Why is it that the curative value of these methods is so little solicited and applied? The question is certainly worthy of discussion, and is at the present moment a particularly opportune one. For the war is unhappily providing large numbers of men crippled and injured in the very fashion in which physical methods claim to be peculiarly serviceable. Here, then, is a large field of opportunity. Yet it would appear from the censures of both of our contributors that our effective resources for utilising this opportunity are of the most limited description. There exists neither the necessary apparatus, nor the organisation, nor the essential directing medical skill; or, at the best, these things exist only on a most restricted scale. The demands of the situation created by the war have in some degree had a stimulating effect, but even now the supplies are quite unequal to the necessities of the case. Nay more, we are told that even apart from the war the curative methods now in question have been and still are largely neglected. They are neither systematically studied, nor effectively taught, nor successfully applied. Such are the terms of the indictment explicit or implied, and assuming the charge to be well-founded the question arises, Who is responsible?

As a general statement of facts, and recognising individual exceptions, it must be allowed that our contributors' descriptions have the virtue of accuracy. It is true beyond doubt that the methods which they advocate have no very wide range of application in this country, and the defect has been made conspicuous by the present emergency. Further, if proceedings having curative value are not set into operation there would seem to be a *primâ facie* case against the medical profession, whose business it is to relieve disability and suffering. The doctors, it must be presumed, either do not know of these methods, or they doubt the value of them, or, from some cause or another, they are hindered from employing them. Perhaps there is a degree of truth in each of these suggestions. No one of them, however, is true in an absolute sense, for physical methods are by no means excluded from medical practice. But it must be admitted that neither the knowledge of them nor the practice of them reaches the level desired by our correspondents, and we are not disposed to deny that the level defined by these gentlemen is a not unreasonable one. If there is neglect there must be some reason for it, as no one can believe that the medical profession out of mere wilfulness or stupidity refuses to employ methods which have a definite curative value. The first step in the removal of hindrances is to secure their definition, and to this extent perhaps the present article may have some value. At all events, it proposes to state in a frank fashion where difficulties lie, and to leave to others the more arduous duty of clearing the obstacles out of the way.

First, there is the position beyond question that the use of physical methods of treatment does not occupy any systematic position in the medical curriculum. The student hears vaguely about massage and electricity, but there is little definite instruction in the therapeutic use of these agencies; and even in the occasional instances in which such instruction is provided it has little attraction to the student, seeing that the knowledge thus provided is not at all likely to prove of service in his qualifying examination. Of course, it is easy to say that instruction of this order ought to be included in the compulsory curriculum. But this in practical terms means both time and expense. The curriculum is already crowded with subjects. An extension of, say, another year would seriously raise the expense of a medical education, which already many regard as out of proportion to the income gained by the average practitioner. Unless a reasonable proportion is kept between the outlay on education and the financial prospects of practice the numbers of those entering the profession will decline, and the public will thus be insufficiently

**PHYSICAL METHODS IN DEFORMITY AND DISEASE**—(*continued*).

supplied with doctors. No doubt it is a very desirable thing that medical students shall be made efficient in all possible directions, but the question of expense here, as elsewhere, asserts itself. There is so much time and so much money for medical education, and these cannot be arbitrarily increased. Hence, those who wish to add new subjects to the curriculum must be prepared either to displace some of the existing ones or to accept the responsibility of increasing the expense of a medical training; and neither of these enterprises is an inviting one.

A second hindrance to the wider application of physical methods of treatment is that not a few of these methods have suffered from the excessive praises of interested or injudicious friends. In certain conditions they all, let it be allowed, have their value, and it is probable that this value is by many practitioners imperfectly appreciated. But there is another side to the picture. Claims of the most extravagant and absurd character have been attached to many of them, and they have been exploited and utilised for the most sordid ends. One has only to look at the advertisement columns even of the most respectable of our lay journals to see vainglorious announcements and boasts which are nothing more nor less than impudent and vulgar quackery. Electricity, baths, gymnastics, massage, diet, all lend themselves to the purposes both of the amateur chatterer and of the money-making quacksalver. Strictly speaking, of course, the philosophic mind should not be influenced in its judgment of the agency by the abuse to which silly or vile persons prostitute it. But human nature being what it is, there is hardly room for surprise in the disinclination on the part of some members of the medical profession to have any association with methods which are so often mere aids to humbug and deceit. When pretence is in excess of reality there is a risk that even the measure of the real which exists should come under suspicion. It is true that the pretence in the matters here discussed is not always the outcome of deliberate fraud, but is merely a consequence of ignorance and of an untrained judgment. This, however, hardly helps the responsibility of the medical profession. Only too often the doctor learns, frequently from public announcements in the press, that some person who is a useful and competent teacher of, say, physical exercises, proposes to undertake, and does undertake, the diagnosis and treatment of disease, though he has had no medical training, and is consequently unaware of the nature of the responsibility he incurs. How is it possible for a medical practitioner to advise patients to place themselves in the hands of such men? To do so in existing circumstances would be to recognise that it is a safe and reasonable thing to accept medical advice and direction from persons ignorant of the very essentials of the art and science of medicine. If members of the public like to accept advice of this order on their own initiative and choice they are free to do so, but the medical practitioner, who knows the risks of such a proceeding, cannot be expected to endorse the proposal. That the risk is not always realised, or even that it is only occasionally realised, does not touch the question. The risk exists, and the practitioner knows that it exists; in a decision to incur it he can therefore have neither part nor lot. Similarly, it is nothing to the purpose that good results may often be claimed for the methods of treatment so administered. There is no need to challenge or to question these results. Nor is it necessary to the argument to deny that serious disasters are only exceptional and occasional. Of these latter little is heard by the general public, but doctors know of their occurrence, and cannot therefore accept any responsibility for a policy which may involve them. Here, indeed, is the main issue so far as the medical profession is concerned. There is no desire to limit the freedom of persons to choose for themselves, but the responsibility for such choice must rest with those who make it. Often, perhaps, the event seems to justify the decision, but it still remains true that winning tricks is not proof of good play. Without question many of these physical methods have close and unhappy associations with humbug, pretence, and ignorance, and this is one of the reasons why the medical profession is apt to look upon them with suspicion and to fail to recognise the degree of value which they undoubtedly possess. Were it possible to detach them from these associations one of the obstacles to their more general adoption would be removed. The task will not be an easy one, but it must be faced if anything like a wide success is to be attained.

A further practical hindrance to the wider use of physical methods as forms of curative treatment is the expense which they involve. It is a blunt statement, but nevertheless true, that many patients, probably the majority, cannot afford the charges which are a necessary part of these methods. This is not to say that these charges, in view of the expenses incurred, are excessive. Apparatus of an elaborate character is necessary, space is necessary, and trained assistants are necessary. Often the treatment means a daily *séance* lasting for an hour or more and extending over weeks or months. There is, in short, both capital and daily outlay on a large scale, and as an inevitable consequence the fees asked from the individual patient are not trifling in amount. Again, many of the conditions to which these methods are applicable are very chronic and obstinate in their course, and even when a favourable issue may be regarded as morally certain—and this is not invariably the case—patients of moderate means, that is the majority of patients, may well find the enterprise beyond their means. In reference to the present position of the injured and crippled soldiers this objection would not appear to apply, for of course the expense of the treatment is undertaken by the State. But the money influence counts even here. For we are told that one of the difficulties of securing the desired methods is the limited number of persons trained in their application. Surely one of the causes of this limitation, probably the main cause, is that in the ordinary

**PHYSICAL METHODS IN DEFORMITY AND DISEASE**—(*continued*).

conditions of national life the opportunity of making a livelihood by these methods is but a limited one, and it is limited because only a relatively small number of patients come within the area of these forms of treatment. The hindrance must arise from one or other of two causes. Either the cases suitable for such treatment are relatively few or, though the cases are suitable, the patients cannot afford the expense. The advocates of physical methods will not allow the first of these propositions, and we are not in the least inclined to disagree with them. They are, therefore, shut up to the second conclusion, and must recognise the financial aspects of the subject as occupying no unimportant place among the present hindrances to their ambitions.

The position may be illustrated by reference to the curative value of climates—one of the subjects which Dr. Fortescue Fox rightly includes in his scheme of treatment by physical methods. He advocates the systematic and scientific study of climate as a therapeutic agency and complains—and no doubt with justification—that it receives little recognition at the hands of the great body of the medical profession. Whether he would make the subject a part of the compulsory medical curriculum or not, we do not know, but he advocates its recognition among those taught by University professors and has done his best to establish it in this position. We are not in the least disposed to quarrel with this advocacy, for a University, as its name professes, ought to offer instruction in all knowledge which can be set forth in a systematic form and order. But we are sure Dr. Fox will allow that climate considered from this point of view is a most involved and intricate topic, and great as may be his difficulty in finding experts in physical orthopædics the task is a trifling one compared with the ambition to set up Professors of Climatology in the British Universities, if, that is, such professors are to do anything more than recite a set of rules founded on a more or less exact series of empirical observations. But suppose this preliminary difficulty overcome, is it to be expected that medical students in any appreciable numbers will spare time for attendance on lectures on climate? We think not, and we fancy that if the Professor of Climatology has to depend on students' fees he will, as an individual problem, be more concerned with food than with climate. All this is not to say that climate is not a useful and even an important agent in a complete and well-ordered therapeutic scheme. On the contrary, let it be freely allowed that its value in this respect is a high one, that the subject is one which ought to be more fully explored, and that the foundation of a University class for dealing with it is an admirable proposition if—and the condition is an essential one—suitable teachers and becoming endowments can be secured. Yet when all this is done there will be no great change in the present position so far as the great majority of medical practitioners and suffering patients are concerned, because as a practical measure the employment of climate for curative purposes is for most persons severely conditioned by economic limitations. The values may be great, undoubted, and beyond challenge, and they may be available to the fortunate few. But they are out of reach of the great majority for the simple reason that they cost more than the patient can afford. Necessarily this position has its influence on medical studies and medical practice. What the patient cannot afford, it is useless for the doctor to recommend. What the doctor recognises as impossible for his patients he will not find time to study. Of course there are exceptions, but these exceptions occupy only a limited part of the field of general practice as this has to be conducted under existing social and economic conditions. This is the plain, hard fact against which a plea for the general study of climate as a scientific therapeutic agent by medical students and practitioners breaks itself in vain. The occasional individual provided with enthusiasm and opportunities and counting on a *clientèle* which is rich in this world's goods will answer to the call, but for the majority the question is outside the realm of practical politics; and neither the reorganisation of knowledge nor the establishment of professorial activities will touch the position, for the plain and simple reason that it is fixed and determined by the economic necessities of the patient. The patient cannot afford the treatment; why then should the busy doctor spend time in studying its possibilities? Climate, we admit, is an extreme case, but what is true here applies in some degree to such proposals as massage, electricity, baths, exercises, and the rest. The expense they entail limits the field within which as a matter of actual experience they are able to be employed, and hence, very naturally, medical practitioners do not find time or inclination for an exact study of methods which circumstances forbid them to advise. Unless the position can be mended in this respect there can be little ground for expecting any wide extension of physical methods as forms of treatment available for others than rich and well-to-do patients. The expenses associated with the cultivation of climatological treatment can hardly be reduced except in so far as it can be shown that advantages credited to distant stations may equally be found nearer home. But by organisation and the establishment of properly controlled institutions other physical methods could certainly be made both more accessible and less costly. This question of expense is a fundamental and vital one, and there is no possibility of any large success apart from it. To it the advocates of the extension of physical methods must needs address themselves. Perhaps their very creditable and praiseworthy enthusiasm for therapeutic achievements may incline them to be neglectful of more mundane considerations, and this is one of the reasons why we press the financial aspect of the matter upon their attention. Just now, as the State foots the bill, they have a great opportunity of showing what these physical methods can accomplish. But ere long the public purse will be closed, and yet the need for money will remain. Hence the necessity of contemplating a future in which State subsidies will no longer be

**PHYSICAL METHODS IN DEFORMITY AND DISEASE**—(*concluded*).

available and of devising schemes to meet a less elastic position.

There are other demands to which a brief reference may be made. One of these is a more exact knowledge of the classes of disabilities and deformities in which physical methods of treatment are of value, and of the extent to which these enterprises must be pursued in order to produce a maximum benefit. If possible, too, it would be a great gain to define for each method its specific virtue and opportunity, so that each may receive its due position in the general therapeutic scheme. Take, for example, the question of baths possessing various physical and chemical properties. How far do these several properties claim specific therapeutic virtues? Are the elaborate developments which they boast essential, or may the same results be reached by less costly and less complicated forms? Similar questions may be advanced in reference to the various forms of massage. In the manuals dealing with the subject great stress is laid upon the special capacities of each variety in relation to particular curative ends. Is there a well-established basis for such claims? We do not ask these questions in any spirit of captious criticism. Rather we have every sympathy with those who are at present concerned with the application of physical methods on a large scale for the benefit of men who have fought and suffered for their country. Our hope is that this experience will be made of permanent value in general medical practice, and our statement of obstacles and difficulties is prompted solely by the desire that these may be recognised as existing facts and that due steps may be taken to overcome or remove them. The methods themselves, we doubt not, have many virtues. But at present they need more precise definition, and this both in a scientific and in a practical sense.

